# Using antibiotic-loaded bone cement for a patient with deep sternal wound infection after cardiac surgery

**DOI:** 10.1093/icvts/ivab332

**Published:** 2021-11-24

**Authors:** Xia Jiang, Guoqing Jiao, Mingqiu Li, Xiaosong Rong

**Affiliations:** Department of Cardiovascular Surgery, Wuxi People's Hospital/Wuxi Affiliated Hospital of Nanjing Medical University, Wuxi, China

**Keywords:** Antibiotic-loaded bone cement, Sternal dehiscence, Deep sternal wound infection

## Abstract

A 67-year-old male presented with sternal dehiscence following open cardiac surgery. Extensive debridement and attempted closure failed, and the wound had since been managed with vacuum-assisted closure therapy, with little progress. We treated him with antibiotic-loaded bone cement to repair the wound defect. After 3 weeks, the wound healed with excellent result. To our knowledge, this is the first report of antibiotic-loaded bone cement for deep sternal wound infection.

## INTRODUCTION

Deep sternal wound infection (DSWI) is a severe complication after cardiac surgery. The incidence of DSWI ranges from 0.2% to 3% depending on multiple risk factors related to the patient, surgeon and procedure. Despite adequate surgical and medical treatment, DSWI causes longer hospital stays and increases cost, with associated mortality ranging from 1.1% to 19% [1].Therefore, it is important to have a reliable technique to treat this kind of infection.

## CASE REPORT

**Figure 1:**
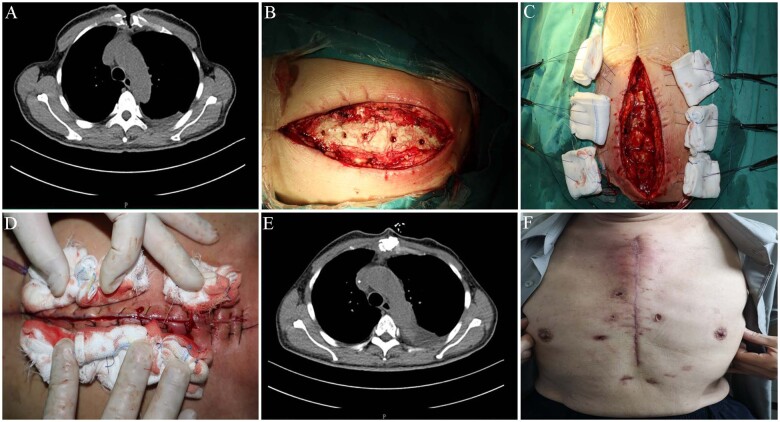
Surgical technique. (**A**) A computed tomography scan prior to the therapy showing sternal non-union. (**B**) Surgical debridement and antibiotic-loaded bone cement covering the sternum defect. (**C**) The entire layer of the skin and the subcutaneous tissue were relaxedly sutured without significant tension. (**D**) Deep sternal wound infection–associated defect after reconstruction and skin suture. (**E**) Computed tomography scan demonstrating the complete sternal healing following 3 weeks of the surgery. (**F**) Healed sternal wound at the 3-week postoperative recovery.

We report a case of DSWI with painful sternal instability 2 weeks after aortic valve and mitral valve replacement through a median sternotomy in a 67-year-old Chinse man. His past medical history was significant for diabetes and chronic renal insufficiency. Vancomycin- and linezolid-sensitive *Staphylococcus aureus* were found in the wound and bone. A thorough mediastinal wash out and surgical debridement was the first step of sternum reconstruction. Next, any infected tissues (the incision and surrounding soft tissues, granulation tissue, dead bone tissue) and steel wire or sutures were removed until normal bone tissue with a good blood supply was reached. After surgical debridement, the antibiotic impregnated cement (PALACOS MV^Ⓡ^+G bone cement, Heraeus, Heraeus Medical GmbH, Wehrheim, Germany) was prepared by combining a 40-g bag of cement with 2 g of vancomycin. The sternal defect was filled with an appropriate amount of ALBC to provide immediate stability. Subsequently, the pectoral muscle and subcutaneous tissue were mobilized from the chest wall. Finally, the entire layer of the skin and the subcutaneous tissue were sutured without significant tension. The drainage tube was placed between the bone cement and the muscle flap (Fig. 1). Fluid samples were obtained for vancomycin concentration measurement from drainage tube. Twenty-four hours after operation, local vancomycin concentration was 450 mg/l and it was 383 mg/l after 48 h. No surgical complications occurred in the postoperative period, and the patient was discharged from the hospital within 3 weeks of the reconstructive procedure. No infection recurrence was observed during the 12-month follow-up.

## COMMENT

The treatment of postoperative DSWI is a real challenge for surgeons. Conservative treatment with debridement and vacuum-assisted closure are not always successful. Pectoralis, rectus and latissimus flaps and omental grafts are commonest forms of sternal reconstruction. Conventional local muscle grafting may not cover wounds effectively or shows the complications of complete or partial flap loss, haematoma, arm or shoulder weakness and incisional hernia [2].

DSWI is invariably accompanied by varying degrees of sternal dehiscence and mediastinitis. Therefore, treatment principle is inclusive of thorough debridement, dead space filling, maintaining sufficient drainage, improving sternal integrity and applying sufficient sensitive antibiotics. Poly-methyl-methacrylate bone cement has been widely recognized as one of most stable antibiotic carriers and is characterized by beneficial functional properties, such as easy processability, fast polymerization, favourable mechanical properties and high biostability in the human body[3, 4]. Aiming at the infection and sternal instability problems of DSWI, we applied the ALBC to our sternal reconstruction system. It provided excellent fixation effect of the sternal wound, with no pathological movement of the chest wall during breathing or activity, no signs of respiratory insufficiency. The effective therapeutic serum concentration of vancomycin is within the range (10–15 mg/l). Local vancomycin concentration sustained release by ALBC, which was largely exceeded (>30-fold) after 24 h and for at least 48 h (>25-fold), ensured the inhibitory of bacteria. Bone cement was used as a carrier to provide high local antibiotic concentrations. Although systemic complications of ALBC spacers are limited, a systematic evaluation has shown that significant antibiotic absorption can occur and the incidence of acute kidney injury may average as high as 5% [5]. Non-detectability of vancomycin in the blood guaranteed the absence of toxicity in the perioperative period. At the same time, serum creatinine and blood urea nitrogen did not increase compared with the first debridement operation.

We believe that this is the first report of such a technique being used in the treatment of a DSWI. The combination of excellent mechanical properties and sustainable drug delivery efficiency demonstrates the potential applicability of ALBC for cardiac surgery to treat DSWI.

**Conflict of interest:** none declared.

## Reviewer information

Interactive CardioVascular and Thoracic Surgery thanks Roman Gottardi, Julian E. Losanoff and the other, anonymous reviewer(s) for their contribution to the peer review process of this article.
